# COVID-19 and Precarious Employment: Consequences of the Evolving Crisis

**DOI:** 10.1177/0020731420986694

**Published:** 2021-01-11

**Authors:** Nuria Matilla-Santander, Emily Ahonen, Maria Albin, Sherry Baron, Mireia Bolíbar, Kim Bosmans, Bo Burström, Isabel Cuervo, Letitia Davis, Virginia Gunn, Carin Håkansta, Tomas Hemmingsson, Christer Hogstedt, Johanna Jonsson, Mireia Julià, Katarina Kjellberg, Bertina Kreshpaj, Wayne Lewchuk, Carles Muntaner, Patricia O’Campo, Cecilia Orellana, Per-Olof Östergren, Eva Padrosa, Marisol E. Ruiz, Christophe Vanroelen, Emilia Vignola, Alejandra Vives, David H. Wegman, Theo Bodin, All Members of the PWR Study Consortium

**Affiliations:** 1 Unit of Occupational Medicine, The Institute of Environmental Medicine, 27106 Karolinska Institutet, Stockholm, Sweden; 2 Department of Social and Behavioral Sciences, Richard M. Fairbanks School of Public Health, 10668 Indiana University-Purdue University Indianapolis, Indiana, USA; 3 Center for Occupational and Environmental Medicine, Stockholm County Council, Stockholm, Sweden; 4 Barry Commoner Center for Health and the Environment, Queens College, 2009 City University of New York, New York City, USA; 5 Health Inequalities Research Group, Employment Conditions Knowledge Network (GREDS-EMCONET), Department of Political and Social Science, 16770 Universitat Pompeu Fabra, Barcelona, Spain; 6 Johns Hopkins University – Universitat Pompeu Fabra Public Policy Center, Barcelona, Spain; 7 Interface Demography, Department of Sociology, 70493 Vrije Universiteit Brussel, Brussels, Belgium; 8 Equity and Health Policy Research Group, Department of Global Public Health, Karolinska Institutet, Sweden; 9 Lawrence S. Bloomberg Faculty of Nursing and Dalla Lana School of Public Health, 70379 University of Toronto, Toronto, Canada; 10 Department of Working Life Science, Karlstad University, Sweden; 11 Department of Public Health Sciences, Stockholm University, Sweden; 12 School of Labour Studies, McMaster University, Hamilton, Ontario, Canada; 13 Social Medicine and Global Health, Department of Clinical Sciences Malmö, 5193 Lund University, Sweden; 14 Department of Public Health, 28033 Pontificia Universidad Católica de Chile, Santiago de Chile; 15 Department of Community Health & Social Sciences, CUNY Graduate School of Public Health & Health Policy, New York, USA; 16 Department of Public Health, Pontificia Universidad Católica de Chile, CEDEUS, Santiago de Chile; 17 14710 University of Massachusetts Lowell, Lowell, USA

**Keywords:** COVID-19, employment, new economy, pandemic, precarious employment

## Abstract

The world of work is facing an ongoing pandemic and an economic downturn with severe effects worldwide. Workers trapped in precarious employment (PE), both formal and informal, are among those most affected by the COVID-19 pandemic. Here we call attention to at least 5 critical ways that the consequences of the crisis among workers in PE will be felt globally: (*a*) PE will increase, (*b*) workers in PE will become more precarious, (*c*) workers in PE will face unemployment without being officially laid off, (*d*) workers in PE will be exposed to serious stressors and dramatic life changes that may lead to a rise in diseases of despair, and (*e*) PE might be a factor in deterring the control of or in generating new COVID-19 outbreaks. We conclude that what we really need is a new social contract, where the work of all workers is recognized and protected with adequate job contracts, employment security, and social protection in a new economy, both during and after the COVID-19 crisis.

The world of work, barely resuscitated after the 2008 Great Recession, is facing an ongoing pandemic and an economic downturn with severe effects on workers worldwide. According to the International Labour Organization (ILO), as of September 2020, 94% of the global workforce was affected by mandatory or recommended workplace closures, and millions of workers faced layoffs and reductions in their working hours.^1^ All told, ILO estimates that between 8.8 and 35 million additional people will be in work-poverty worldwide by the end of 2020.^2^ These labor market effects are not distributed equally. The relative decline in employment is greater for women than for men and for lower caste workers in all countries.^1^ Another inequality is seen across employment arrangement: Workers trapped in precarious employment (PE), both formal and informal, are among those most affected by the COVID-19 pandemic.^3^ Now is the time to address the huge inequalities in our global labor market. As Guy Ryder, ILO director general, said, “The crisis has uncovered the huge decent work deficits that still prevail in 2020 and shown how vulnerable millions of working people are when a crisis hits.”

PE is commonly defined as jobs that accumulate several unfavorable features of employment quality, such as employment insecurity (e.g., contractual temporariness, contractual relation insecurity, underemployment, and multiple job holding), inadequate income, and limited rights and protection (e.g., lack of unionization, social security, regulatory support, and workplace rights).^4,5^

PE is an important social determinant of health, associated with a multitude of poor health outcomes.^6,7^ PE is more common in already disadvantaged or vulnerable groups, which generates systematic, unfair, and avoidable differences in health.^6^ Workers in PE often find themselves in situations where their governments and employers do not provide access to sufficient social and health protections. Globally, only 45% of the population is covered by at least 1 social protection benefit, which means that 55% is completely unprotected.^8^

Here we call attention to at least 5 critical ways that the consequences of the crisis among workers in PE will be felt globally.

First, PE is likely to increase due to the crisis of the COVID-19 pandemic because the rise of unemployment will undoubtedly be followed by an increase in PE (a phenomenon observed in the 2008 financial crisis). As a result of expanded PE, workers are likely to face long-term labor market disadvantages. One example is the group referred to as the “lockdown generation”: Young workers who have suffered disruptions in education and training being pushed into insecure and low-wage jobs with reduced working hours.^9^

Second, workers in employment that was already precarious before the pandemic risk becoming even more precarious: With limited bargaining power, they will be more vulnerable to unfair treatment, abuse, and exploitation.

Third, workers in PE may face unemployment without being officially laid off—for example, by not having contracts renewed or seeing a reduction in working hours to zero—and thus many will not be eligible for unemployment benefits.

Fourth, workers in PE may experience barriers in accessing health care, because many lack adequate health insurance or access to health insurance,^10^ together with difficulties in maintaining adequate housing conditions and accessing adequate amounts of food, given reduced incomes. These stressors and dramatic life changes may lead to a rise in diseases of despair, such as substance use disorders, mental health problems, and suicide attempts.^11,12^ Moreover, workers in PE, who are often unable to work safely from home, will experience poorer work–life balance, be exposed to greater risk of household virus spread, and suffer family conflicts.

Fifth, PE might be a factor in deterring the control of or in generating new COVID-19 outbreaks. Because workers in PE often lack access to paid sick leave, they will be forced to work while sick to avoid losing income or a job, further accelerating the unequal spread of COVID-19.^13^ In addition, many workers in PE have continued to work in environments that lack adequate virus control and safety measures.^14^ These factors increase the risk of infection among workers, their families, and the broader public.

Now, possibly more than ever before, there is an urgent need for equitable and inclusive policy responses to guarantee Article 25.1 of the Universal Declaration of Human Rights and to prioritize decent work in the Sustainable Development Goals agenda. Just as everyone has the same universal rights, having universal social protections would mitigate the impact of this pandemic and prepare us to meet the next one with greater solidarity. What we really need is a new social contract, where the work of all workers is recognized and protected with adequate job contracts, employment security, and social protection in a new economy, both during and after the COVID-19 crisis.

## Supplemental Material

sj-pdf-1-joh-10.1177_0020731420986694 - Supplemental material for COVID-19 and Precarious Employment: Consequences of the Evolving Crisis Click here for additional data file. Supplemental material, sj-pdf-1-joh-10.1177_0020731420986694 for COVID-19 and Precarious Employment: Consequences of the Evolving Crisis by Nuria Matilla-Santander, Emily Ahonen, Maria Albin, Sherry Baron, Mireia Bolíbar, Kim Bosmans, Bo Burström, Isabel Cuervo, Letitia Davis, Virginia Gunn, Carin Håkansta, Tomas Hemmingsson, Christer Hogstedt, Johanna Jonsson, Mireia Julià, Katarina Kjellberg, Bertina Kreshpaj, Wayne Lewchuk, Carles Muntaner, Patricia O’Campo, Cecilia Orellana, Per-Olof Östergren, Eva Padrosa, Marisol E. Ruiz, Christophe Vanroelen, Emilia Vignola, Alejandra Vives, David H. Wegman, Theo Bodin and All Members of the PWR Study Consortium in International Journal of Health Services
