# Supplementary material for: COVID-19 and Precarious Employment: Consequences of the Evolving Crisis
Source: Int J Health Serv. 2021 Jan 11;51(2):226–8. doi: 10.1177/0020731420986694 (PMC8114423; doi:10.1177/0020731420986694)
Supplement: sj-pdf-1-joh-10.1177_0020731420986694 - Supplemental material for COVID-19 and Precarious Employment: Consequences of the Evolving Crisis [file sj-pdf-1-joh-10.1177_0020731420986694.pdf]

Brief summary of the PWR consortium:

Precarious Work Research (PWR) is a research program on non-standard and precarious employment. We are an international group of researchers in Sweden (Karolinska Institutet, Stockholm University, Karlstad University, Lund University), Belgium (Vrije Universiteit Brussel), Spain (Universitat Pompeu Fabra), Chile (Pontificia Universidad Católica de Chile), USA (University of Massachusetts Lowell, Indiana University-Purdue University Indianapolis, City University of New York) and Canada (University of Toronto, McMaster University). The program is funded by the The Swedish Research Council for Health, Working Life and Welfare Forte. More information can be found in: https://precariousworkresearch.org/
